# Fibroblast Interaction with Different Abutment Surfaces: In Vitro Study

**DOI:** 10.3390/ijms21061919

**Published:** 2020-03-11

**Authors:** Luigi Canullo, Tullio Genova, Esperanza Gross Trujillo, Guillermo Pradies, Sara Petrillo, Maurizio Muzzi, Stefano Carossa, Federico Mussano

**Affiliations:** 1 Private Practice, Via Nizza, 46, 00198 Rome, Italy; 2 Department of Life Sciences and Systems Biology, University of Torino, 10126 Turin, Italy; tullio.genova@unito.it; 3 CIR Dental School—Department of Surgical Sciences, University of Torino, Via Nizza 230, 10126 Turin, Italy; stefano.carossa@unito.it (S.C.); federico.mussano@unito.it (F.M.); 4 Department of Buccofacial Prosthesis, University Complutense, 28040 Madrid, Spain; egross@ortizvigon.com (E.G.T.); gpradies@odon.ucm.es (G.P.); 5 Department of Molecular Biotechnology and Health Sciences, University of Rome III, 00133 Rome, Italy; sara.petrillo@unito.it; 6 Department of Science, University of Rome III, 00133 Rome, Italy; maurizio.muzzi@uniroma3.it

**Keywords:** abutment integration, abutment characteristics, dental implant abutment, in vitro study, fibroblast, bioactivation, plasma of argon, UV light

## Abstract

Background: Attaining an effective mucosal attachment to the transmucosal part of the implant could protect the peri-implant bone. Aim: To evaluate if chair side surface treatments (plasma of Argon and ultraviolet light) may affect fibroblast adhesion on different titanium surfaces designed for soft tissue healing. Methods: Grade 5 titanium discs with four different surface topographies were subdivided into 3 groups: argon-plasma; ultraviolet light, and no treatment. Cell morphology and adhesion tests were performed at 20 min, 24 h, and 72 h. Results: Qualitative observation of the surfaces performed at the SEM was in accordance with the anticipated features. Roughness values ranged from smooth (MAC Sa = 0.2) to very rough (XA Sa = 21). At 20 min, all the untreated surfaces presented hemispherical cells with reduced filopodia, while the cells on treated samples were more spread with broad lamellipodia. However, these differences in spreading behavior disappeared at 24 h and 72 h. Argon-plasma, but not UV, significantly increased the number of fibroblasts independently of the surface type but only at 20 min. Statistically, there was no surface in combination with a treatment that favored a greater cellular adhesion. Conclusions: Data showed potential biological benefits of treating implant abutment surfaces with the plasma of argon in relation to early-stage cell adhesion.

## 1. Introduction

The long-term success of dental implants depends, among other factors, on the establishment and maintenance of crestal bone levels in relation to the formation of a soft tissue barrier [1]. The peri-implant soft tissues have become a major concern in recent years as the presence of an effective mucosal attachment at the transmucosal part of the implant can provide the implant with protection of the peri-implant bone from bacteria contamination and oral environment, preventing peri-implant pathologies [1,2,3]. This aspect is of particular relevance in the aesthetic area, where the stability of gingival margin and papilla is related to the maintenance of crestal bone [3].

Osseointegration and cell adhesion are both influenced by the surface properties of dental implants and implants abutments. These properties include topography, roughness, chemistry, charge, and hydrophilicity [4,5,6]. Therefore, there has been a tendency to investigate methods to change surface chemistry to promote faster osseointegration and stability of the implants as well as to achieve a rapid fibroblast and epithelial cell adhesion and proliferation to the abutments [7,8,9,10].

Due to the lack of attachment of connective tissue fibers to the implant surface, implant collars have been modified including a microtextured portion to enhance soft tissue attachment to the implant cervical area [11,12]. On the other hand, some experimental and clinical studies have shown physical attachment of the connective tissue to the laser micro-texturized collar of dental implants with less bone loss than smooth collars [9,13,14,15]. By increasing the surface energy, and consequently the surface hydrophilicity, one can enhance the interaction between the implant and the biological environment, improving cell adhesion [4]. To promote the hydrophilicity of the titanium surfaces the following methods have been described: exposure to ultraviolet light (UV) [16], alkali therapy [17], or plasma processing [18].

Plasma treatment can decontaminate surfaces without modifying their topography [19]. It is also able to increase surface energy, obtaining more hydrophilic surfaces, which may increase the capacity of the titanium oxide layer to interact with proteins and cells of surrounding tissue improving cell adhesion [18,20]. Experimental studies have indicated that non-thermal plasma of argon treatment has a positive effect on osteoblast adhesion and spreading [18,21] and protein adsorption on different common titanium surfaces [21].

To provide a stable relation between soft tissue and crestal bone around dental implants is critical for achieving and maintaining clinical success. As a consequence, there is a need for investigative methods to enhance biologic surface properties [19]. Although several studies have indicated, so far, that UV light and non-thermal plasma may improve surface reactivity [16,18,20], it is not known whether UV light and plasma treatment are comparable in terms of adhesion when different titanium surfaces designed for soft tissue healing are evaluated.

The aim of this in vitro study was, therefore, to evaluate if plasma treatment or ultraviolet light could affect the adhesion of fibroblasts over different titanium surfaces designed to interface with connective tissue cells.

## 2. Results

### 2.1. Microscopic and Topographic Analysis

**Figure 1:**
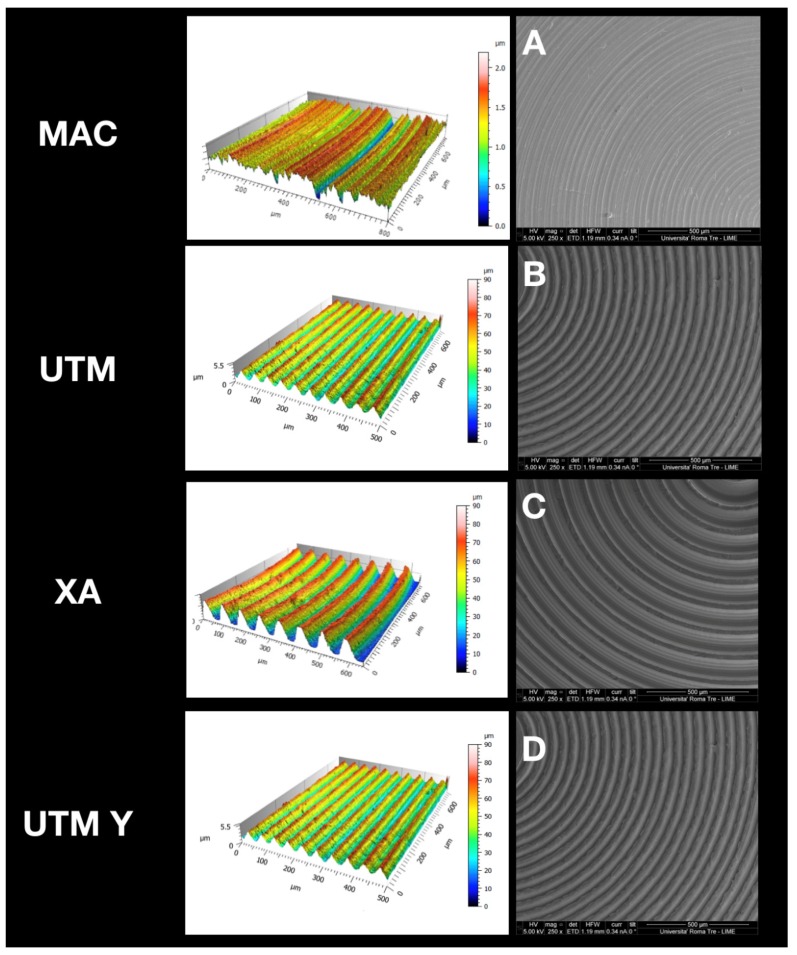
Topographic analysis of the selected surfaces. (**A**): MAC; (**B**): (UTM); (**C**): (XA); (**D**): (UTM Y).

At SEM observation, all surfaces were clean without visible contaminations (Figure 1). Topographic analysis of machined disc MAC highlighted a smooth surface with circular micro-threads with depth lower than 2 µm (Figure 1A), with a Sa mean value of 0.2. The other tested surfaces appeared all grooved because of the peculiar presence of variably deep parallel micrometric sulci (hence the “micro-grooved” pattern). UTM surface presented a particular threading with a triangular profile and pitch of 50 µm (Figure 1B). XA surface (Figure 1C) was more deeply threaded with a pitch of 80 µm and a depth of 50 µm. The Sa mean value of this surface was 21, and its Sdr 119%, consistently with its micro-topography. Although anodized, UTM-Y surface (Figure 1D) resembled that of UTM, whose Sa and Sdr were respectively 0.6% and 2.8%, the same values as UTM.

### 2.2. Wettability

The surfaces were tested for the wetting properties by optical contact angle (OCA) measurements of water drops. As reported in Table 1, MAC showed an average contact angle value of 77°, while UTM and UTM-Y resulted in progressively more hydrophobic (H20 CA° being respectively 82° and 96°). On the contrary, XA was the most hydrophilic of all the surfaces.

### 2.3. Cell Morphology and Scanning Electron Microscope Analysis

**Figure 2:**
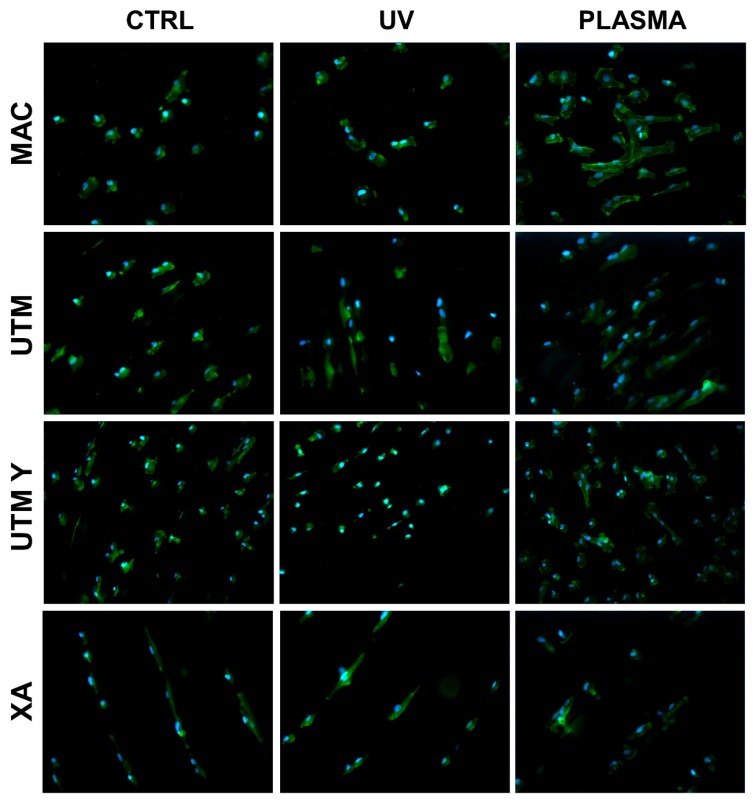
Cell morphology 20 min after seeding at 200X. Cell cytoskeleton was stained by using phalloidin (green) and the cell nuclei were stained by using DAPI (blue).

**Figure 3:**
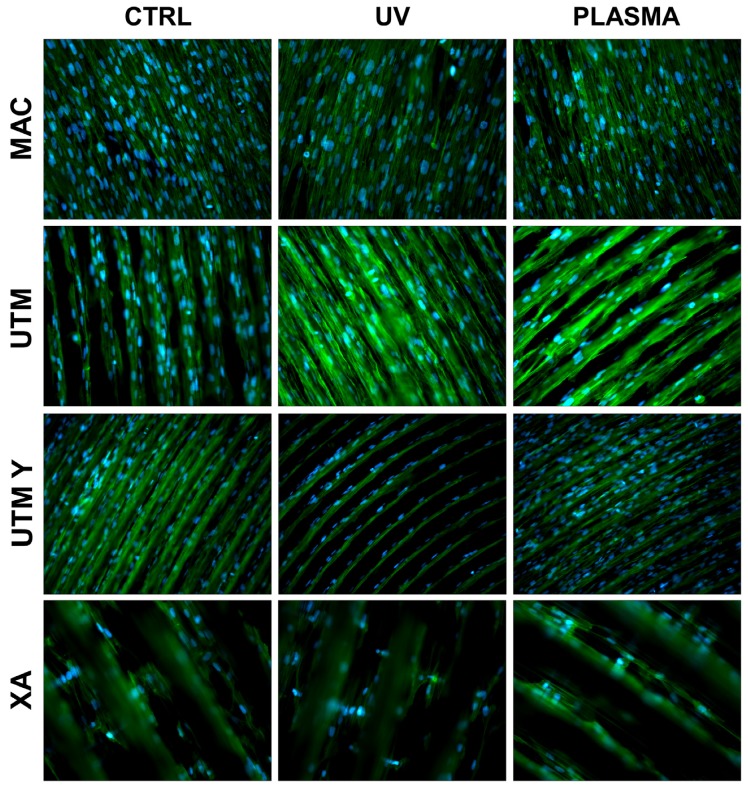
Cell morphology 24 h after seeding at 200X. Cell cytoskeleton was stained by using phalloidin (green) and the cell nuclei were stained by using DAPI (blue).

**Figure 4:**
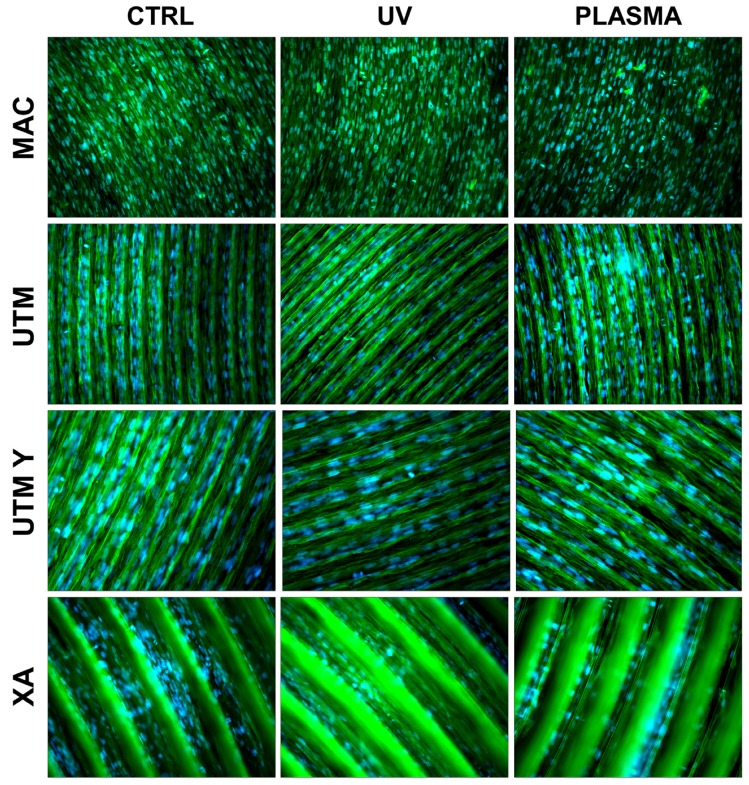
Cell morphology 72 h after seeding at 200X. Cell cytoskeleton was stained by using phalloidin (green) and the cell nuclei were stained by using DAPI (blue).

**Figure 5:**
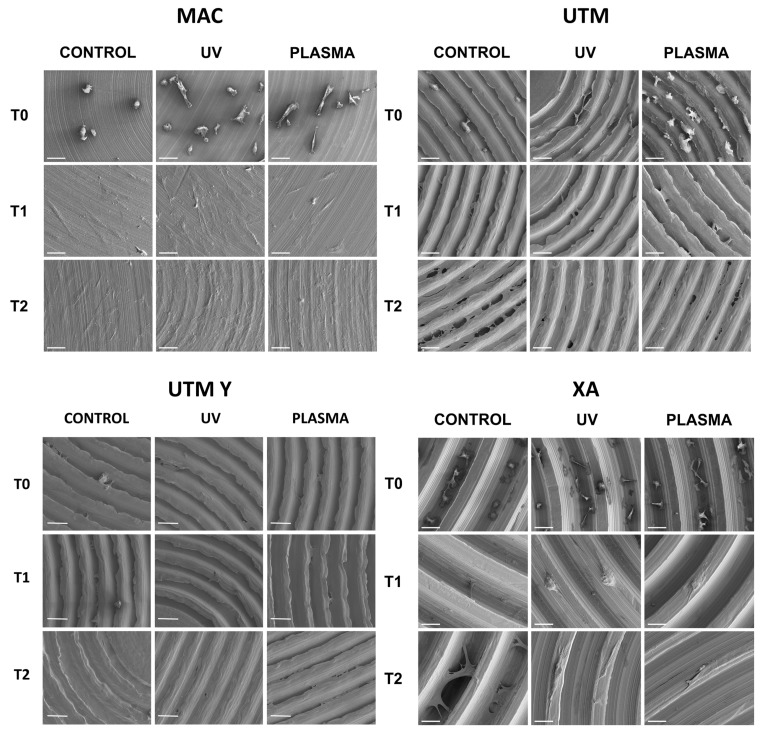
SEM images of cell adhesion at different timing (20 min, 24 h, 72 h) comparing different surfaces.

The behavior of cells on different surfaces at different timepoints are depicted in Figure 2, Figure 3, Figure 4 and Figure 5. The analysis of MAC samples suggests that plasma treatment affected the growth/morphology of the cells only during the first phases of their adhesion, while UV treatment did not exert any effect.

At T0 (20 min), the cells seeded on MAC (Figure 2 and Figure 5) were hemispherical with reduced and delicate filopodia, while, the cells grown on the same plasma-treated surfaces became spindle-shaped with broad lamellipodia and rare filopodia. The effect of plasma of Argon was confirmed also in UTM, UTM-Y, and XA samples: treated discs showed cells with more spread and extended shape compared to the untreated samples (Figure 2 and Figure 5). Notably, in both control and treated UTM discs, the cells were more numerous on the flanks of the grooved surface. In XA control samples the cells were attached almost exclusively to the bottom of the grooved surface and some of them showed a spreading morphology (Figure 2 and Figure 5). In the treated sample, cells were more elongated compared to the control and they appeared more evenly distributed throughout the sample, adhering not only on the bottom but also on the top and the flanks of the grooves.

At T1 (24 h), all samples, both treated and untreated ones, presented abundant and well-developed cells that exhibited a spread morphology and several cellular extensions (Figure 3 and Figure 5). Fibroblasts in treated and untreated samples were comparable, except for a slight increase in cell area in the plasma-treated samples (Figure 3 and Figure 5).

In both control and plasma-treated samples of UTM and UTM-Y, several cells showed a flat, spindle form, and appeared preferentially distributed between the ridges, connecting the flanks of the grooved surface (Figure 3 and Figure 5). Fibroblasts grown on XA control disc appeared as flattened cells, located at the bottom of the ridges, or as elongated cells, between the flanks of the ridges. In the treated samples, instead, many cells grew on the crest of the ridges, showing an unusual morphology (fusiform but markedly swollen in the center), as portrayed in Figure 3 and Figure 5.

At T2 (72 h), all the specimens allowed the same spread morphology with a similar covered area, but exhibited an increased growth compared to T1 samples. At this time point, the quantitative differences previously described between treated and control discs were strongly reduced (Figure 4 and Figure 5). It is worth noting that only on treated XA surfaces, the cells continued to show peculiar spindle morphology and grew on the top of the ridges. On the contrary, in the untreated discs, the cells were flattened and located between the ridges or at the bottom of the groove (Figure 4 and Figure 5).

### 2.4. Cell Adhesion

**Figure 6:**
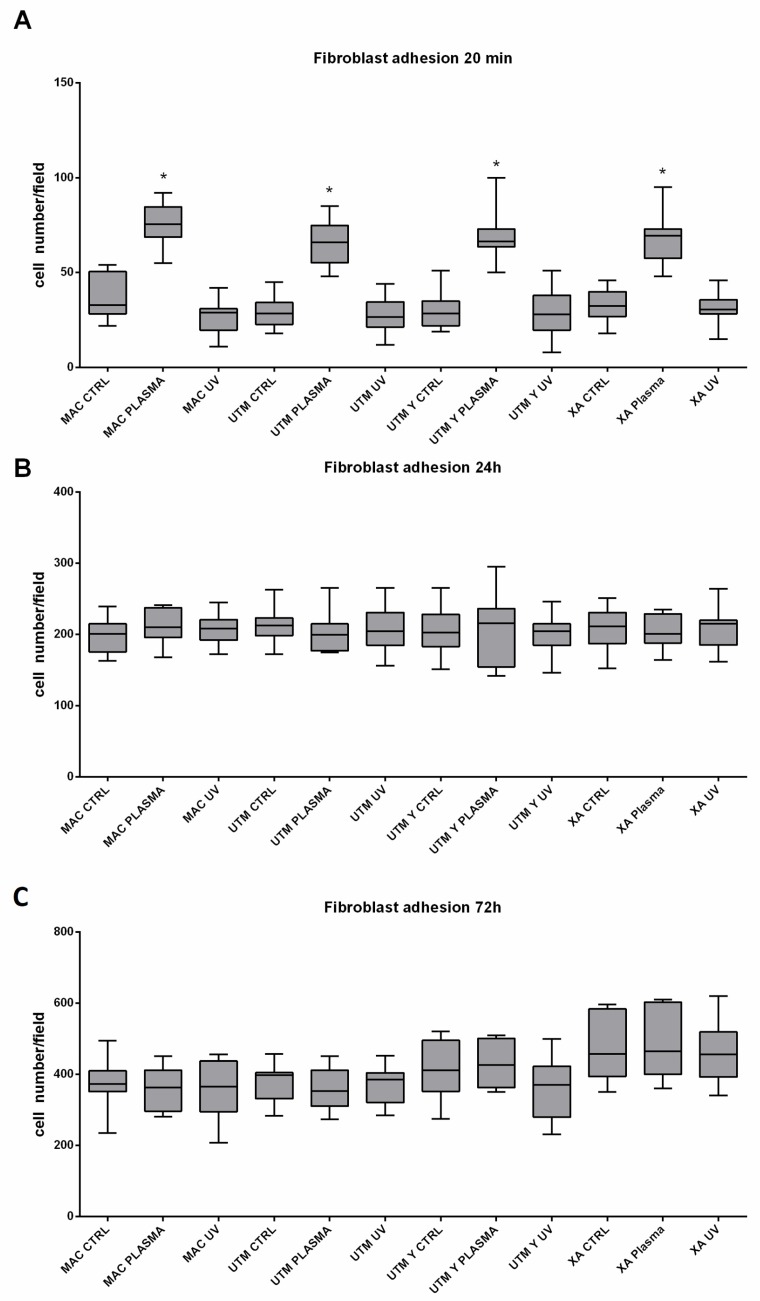
Representation of adherent fibroblast at 20 min (**A**), 24 h (**B**), 72 h (**C**). Statistical analysis was performed using three-way ANOVA with Tukey’s corrections for multiple comparisons. * represents a significant difference versus the relative time point control condition. Surfaces (made of grade 5 Ti): MAC: machined titanium; UTM: ultrathin threaded microsurface titanium; UTM-Y: anodized ultrathin threaded microsurface titanium; XA: deep threaded surface. Treatments: control; no treatment; UV: ultraviolet light; plasma; non-thermal plasma treatment.

As for the surface treatment, at T0, in all different titanium samples, plasma of Argon significantly increased the number of adherent fibroblasts compared to the controls (Table 2 and Table 3; Figure 6). This difference, however, was no longer statistically significant at T1 and T2 (Table 2 and Table 3). Ultraviolet light resulted in less effective than Plasma of Argon. Indeed, at T1, the number of adherent cells was similar to the control and, at T1 and T2, the number of adherent fibroblasts, although higher on UV treated than untreated surfaces, was not different in a statistically significant way (Table 2, Table 3 and Table 4; Figure 6). As regards the surface type, no difference could be detected at any time point (Table 4).

### 2.5. Focused Ion Beam Evaluation of Fibroblasts Layers

**Figure 7:**
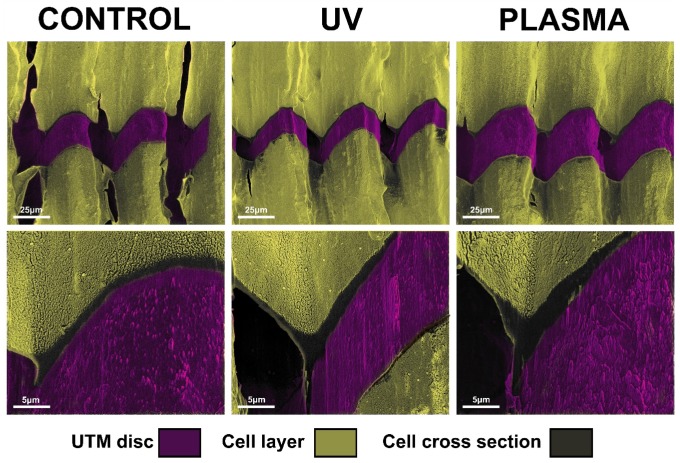
Image of FIB/SEM cross-sections showing the interface between the cells and the surface of untreated, UV treated and plasma-treated UTM discs.

After 72 h, complete coverage of the discs’ surface was observed for the UTM and UTM-Y samples. Hence the FIB column was used to perform a selective ablation of these samples to evaluate the difference in cell layer thickness. The growth pattern of the cells appeared the same in both treated and untreated samples with fibroblasts completely adhering to the top of the titanium crests. However, UTM and UTM-Y discs treated with either plasma or UV displayed a slight increase in cell layer thickness compared to the untreated discs, with an increase ranging from 30% to 50% (Figure 7).

## 3. Discussion

The establishment and maintenance of an efficient soft tissue seal around dental implants and abutments are hallmarks for implant success [22]. The surface properties of implant elements have proven to influence the quality of this mucosal attachment. To improve the interaction between recipient tissues and implant components different surface modifications have been proposed, among which topography, roughness, and chemical qualities have been the research focus [23,24].

While the effects of surface roughness on the bone response have been widely discussed [25,26,27], the scientific data are lesser, with regards to the impact of surface roughness on soft tissue attachment. Some histological investigations in humans and animals support the fact that moderately rough surfaces may favor soft tissue integration [28,29,30]. This observation is in accordance with the present study, where micro-grooved surfaces promoted higher levels of cell adhesion than MAC, although only XA was statistically significant.

Besides roughness, chemical surface modifications such as those obtained by anodization of grade 5 titanium have been suggested to enhance the early biological response of gingival cells when dealing with machined smooth surfaces [31]. The anodization process was used here to modify UTM attaining UTM-Y, endowed with a yellowish color (hence the suffix Y). These micro-grooved and anodized micro-grooved surfaces had identical roughness features, but they differed in terms of wettability (Table 1). The anodization process promoted indeed a slight transition of UTM-Y (H20 CA = 96°) toward the hydrophobic regime compared to UTM (H20 CA = 82°).

Surface energy plays a relevant role among the surface properties of implants components [32]. More specifically, hydrophilicity may promote cell adhesion, being beneficial during the early stage of wound healing [33]. Both UV light and plasma cleaning can increase surface wettability [19]. In this in vitro model, significantly higher values of cell adhesion could be detected due to the plasma treatment at 20 min irrespective of the type of surface. Differences, however, lost their statistical significance with time. Interestingly the wettability of pristine surfaces, albeit quite different, could not affect fibroblast adhesion in a statistically significant way (Table 1). Data here presented are in line with previously reported outcomes evidencing that plasma treatment is capable of enhancing cell adhesion on titanium surfaces, mostly at the early stage [18,34]. On the other hand, no differences were found between the ultraviolet light group and untreated discs in terms of cell adhesion, unlike other studies where UV treatment seemed to increase this parameter [21].

Whether wettability, which is indeed influenced both by surface topography and chemical composition [35], is sufficient to predict the biological outcome is still a matter of debate. For instance, Gittens et al. [36], in a magistral paper of theirs, stated that “available techniques to measure surface wettability are not reliable on clinically relevant, rough surfaces,” and they noticed that the behavior of the cell model used was dependent on its differentiation state. Although these authors were working with lineage osteoblasts, the consideration sheds light on the delicacy of any cell model, fibroblasts included.

In this study, fibroblasts preferentially adhered to the peaks of roughened surfaces. According to Chang et al. [37], one may speculate that fibroblasts reacted to a fibronectin density possibly higher on activated surfaces than the untreated controls, by forming more adhesion complexes. Whatever the mechanism involved at T0, at T2, no differences could be found between treatment groups. A reasonable explanation thereof could be the duration of plasma of argon effects, as this treatment is very powerful, but it tends to be most active in a limited period of time, which may not be detrimental for chair side usage. As pointed out elsewhere [21], another aspect worthy of consideration is the saturation effect owing to rapid cell growth on a small surface like a disc. The present work tried to overcome, at least in part, this usual drawback of in vitro studies recurring to a strong statistical setting. Furthermore, the thickness of cells overgrowing at T2 was considered carefully.

The FIB column ablation allowed to show a slight difference in terms of cell stratification, which may suggest promising results in case of longer observation time-point. A bi-dimensional observation, indeed, may fail to detect “vertical growth” following cell stratification. In the present study, FIB, by ablating part of the cell carpet, allowed a tridimensional observation of cell layers, thus highlighting any possible difference among samples. In particular, UTM and UTM-Y discs were the only samples completely covered with cells at T2 and they displayed a slight increase in cell layer thickness compared to untreated discs, independently of the type of treatment received (UV or Plasma of Argon). This qualitative observation suggested that FIB/SEM might be useful for analyzing cell layering in future studies.

However, the main limitation of this research is, obviously, the very in vitro model. In spite of the positive and encouraging outcomes, this study must be confirmed in vivo and, even more compellingly, recurring to clinical trials. Finally, the selected micro-topographies exemplified three of the most common families adopted on the abutments (smooth, micro-grooved, anodized), but they did not represent all the possibilities available on the market.

## 4. Materials and Methods

### 4.1. Sample Size

A power analysis was performed by referring to a similar preclinical study, which investigated the same topic [38]. Based on these data, mean fibroblast adhesion values of 181 ± 37 and 135 ± 26 at 20 min (*p* = 0.0039) was projected by setting effect size dz = 1.438, error probability alpha = 0.05, and power = 0.95 (1-beta error probability), resulting in 12 samples from each sub-group (G* Power 3.1.7 for Mac OS X Yosemite, version 10.10.3).

### 4.2. Sample Preparation

**Figure 8:**
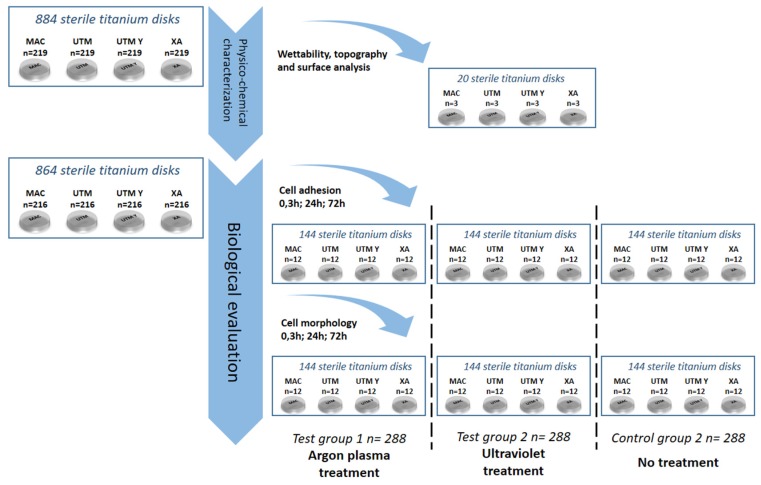
Flow chart of the randomization sequence explaining the study design and the allocation of the samples.

As portrayed in Figure 8, 884 serially numbered, sterile discs (Sweden & Martina), made of grade 5 titanium, with four different surface topographies were used for this study:

machined (MAC);

“micro-grooved” surfaces Ultrathin Threaded Microsurface (UTM);

“micro-grooved” Anodized Ultrathin Threaded Microsurface (UTM-Y);

“micro-grooved” Thin Machined (XA).

All disks had a diameter of 10 mm and a height of 3 mm. After manufacturing, all the titanium discs underwent the same standard cleaning and sterilization procedure that is used for commercial dental implants. Three discs per surface type underwent surface and micro-topography analyses. Two discs per surface type underwent an analysis of wettability. The remaining 864 titanium discs, i.e., 216 per each of the 4 surfaces, were randomly allocated into three sub-groups of 288 samples as follows:

i. Argon plasma treatment at 8 W and atmospheric pressure for 6 min, using a plasma reactor, Plasma R, Diener Electronic GmbH, Ebhausen, Germany, (test group 1, TG1)
ii. UV treatment [Ultra Violet light treatment (Toshiba, Tokio, Japan) for 3 h (15 W) at ambient conditions [intensity: 0.1 mW/cm2 (λ = 360 ± 20 nm) and 2 mW/cm2 (λ = 250 ± 20 nm)] (test group 2, TG2)
iii. No treatment (control group, CG).

Every treatment subgroup counted a total of 72 samples per surface and was further subdivided into either a cell adhesion group (*n* = 36) or a cell morphology group (*n* = 36). Finally, three computer-generated randomization lists (Random Number Generator Pro 2.08 for Windows, Segobit Software, http://www.segobit.com/) were used to randomly allocate the titanium discs into three sub-groups (T0, T1, T2), consisting in an equal number of 12 titanium discs each. All the computer-generated randomization lists were prepared in advance by an external investigator not involved in the study and an independent consultant prepared all of the envelopes/containing numbers for randomization, which were opened immediately before the testing procedures.

### 4.3. Topographic Analysis

Area surface roughness parameters at different sites of the implant were obtained by scanning electron microscope (SEM), using an EVO MA 10 SEM (Zeiss, Oberkochen, Germany). In particular, the Stereo Scanning Electron Microscope (SSEM) technique was used. This approach exploits the basic principle of stereo vision to turn conventional SEM images into three-dimensional surface topography reconstructions. Two images of the same field of view are acquired after eucentric rotation at a given angle. This is obtained by changing the angle between the sample and the electrons source, by tilting the table bearing the sample. The tilting angle is set and controlled by the instrument control software. The recorded incoming data were the couple of images obtained (stereopair), the size of the field of view, and the tilting angle and they were processed using a specific software (Mex 6.0, Alicona Imaging, Chicago, IL, USA).

Three-dimensional images obtained by this process allowed us to measure height profiles or areas, and to calculate the different roughness parameters defined by relevant literature and standards (Table 1). In the present analysis, SEM images used to build-up stereo-pairs were obtained at 2000×. Roughness parameters according to ISO25178 were obtained from reconstructed images of 80 × 110 micrometers area.

### 4.4. Wettability

To assess the wetting properties of the samples, the optical contact angle (OCA) of a sessile water drop (1 μL in volume) was measured through the OCAH 200 (DataPhysic Instruments GmbH, Filderstadt, Germany). The integrated high-resolution camera allowed us to acquire the image of the drop on each specimen, while the drop profiles were extracted and fitted with a dedicated software (SCA20) through the Young–Laplace method. Contact angles between the fitted function and baseline were calculated at the liquid–solid interface [39,40].

### 4.5. Cell Culture

To characterize the biological response in vitro, Normal Human Dermal Fibroblasts were used (NHDF). Fibroblasts were maintained in Dulbecco Minimum Essential Medium (DMEM).

These cells represent an excellent model for studying the dynamics of fibroblast adhesion [31,41,42,43,44] and have similar behavior in culture to the gingival fibroblast despite some differences; indeed, their fundamental characteristics are almost identical [41].

The culture media were supplemented with 10% fetal bovine serum (Life Technologies, Milan, Italy), 100 U/mL penicillin, 100 mg/mL streptomycin, were passaged at subconfluency to prevent contact inhibition and were kept under a humidified atmosphere of 5% CO_2_ in the air, at 37 °C.

### 4.6. Cell Morphology

Cells were seeded on titanium discs (*n* = 648) at a concentration of 10^4^ cells/well in a 48-well plate (BD, Milan Italy) and then kept in growth condition. After 20 min, 24 h and 72 h (T0, T1, T2), the titanium specimens were washed in Phosphate Buffer Saline (PBS) and then the cells were fixed with 4% paraformaldehyde (PFA) in PBS for 15 min. After two washes with PBS, cells were permeabilized with 0.1% Triton X-100 (Sigma-Aldrich) in PBS. Following the manufacturer’s protocol, cells were stained with Rodhamine-Phalloidin (Life Technologies) and 1 uM 4′,6-diamidino-2-phenylindole (Dapi, Life Technologies) to respectively detect the cytoskeleton and the nuclei [45,46]. Image acquisition was made recurring to a Nikon Eclipse Ti-E microscope with 40X objective (Plan Fluor Nikon) [47]. Image analysis [48,49,50] was performed using ImageJ software (ImageJ, U.S. National Institutes of Health, Bethesda, MA, USA, http://imagej.nih.gov/ij/).

### 4.7. Cell Adhesion

Cell adhesion was evaluated on 648 titanium samples using a 48-well plate (BD, Milan, Italy). Cells were detached using trypsin for 3 min, carefully counted, and seeded at 3 × 10^3^ cells/well in 1 mL of growth medium on the different samples. The 48-well plates were kept at 37 °C, 0.5% CO_2_ for 20 min, 24 h and 72 h. Before and after fixation in 4% paraformaldehyde in PBS for 15 min at room temperature, cells were washed two times with PBS and then stained with 1 μM DAPI (Molecular Probes, Eugene, CA, USA) for 15′ at 37 °C to detect cell nuclei. Samples were analyzed using a Nikon Eclipse T-E microscope with a 4X objective. Cell nuclei were then counted by using ImageJ (NIH) software with the tool “Analyze particles” [51,52].

### 4.8. Scanning Electron Microscope/Focused Ion Beam Analysis

To test if in vitro conditions at the longer time points could generate a different cell layering, samples at T2 were dehydrated with a graded ethanol series, air-dried, and secured to an aluminum stub with a conductive adhesive carbon disc. Subsequently, the specimens were sputter-coated with a thin layer (30 nm) of gold using a K550 sputter coater (Emithech, Kent, UK) and examined by the Dual Beam Helios Nanolab 600 (FEI, Hillsboro, state, USA). Micrographs of the samples were acquired detecting secondary electrons, using an operating voltage of 5 kV and an applied current of 0.17 nA

Additionally, the Focused Ion Beam (FIB) column was used to selectively ablate a small region of the cell layer, making it possible to evaluate its thickness and the interaction between the fibroblasts and the titanium surface.

### 4.9. Statistical Analysis

Data were recorded on Excel 2011 data sheet (Microsoft Corporation, Redmond, WA, USA) and analyzed by using Statistical Analysis Software (SAS; Cary, NC, USA) and GraphPad Prism 6 [53,54,55]. The following independent variables were considered: (1) the type of surface, (2) the type of treatment, (3) the time of assessment. The number of fibroblasts on in vitro titanium discs was the primary dependent outcome variable. Data were expressed as means and 95% confidence intervals. To compare the effect of type of surface, type of treatment, time of assessment, and their interaction on the main outcome variable, a general linear model was performed, using three-way ANOVA with Tukey’s corrections for multiple comparisons [56,57].

## 5. Conclusion

Within its limitations, this in vitro study highlighted the capability of micro-grooved surfaces to attract and distribute cells, suggesting the potential biological benefits of treating implant surfaces with the plasma of argon in relation to early-stage cell adhesion. The positive reported outcomes encourage the use of micro-grooved surfaces and bio-activation in in vivo studies.

## Figures and Tables

**Table 1:** Different roughness (Sa, Ra, Sdr, and Sds) and wettability (H20 CA°) parameters for the different surfaces.

Surface	Sa	Ra (μm)	Sdr%	Sds (1/μm^2^)	H20 CA°
MAC	0.2	0.11	0.748	0.0856	77 ± 1.4
UTM	0.6	0.62	2.80	0.0772	81.7 ± 1.6
UTM-Y	0.6	0.62	2.80	0.0772	95.9 ± 6.4
XA	21	5.56	119	0.0239	45 ± 20

Surfaces (made of grade 5 Ti): MAC: machined titanium; UTM: ultrathin threaded microsurface titanium (micro-grooved); UTM-Y: nodized ultrathin threaded microsurface titanium (micro-grooved); XA: deep threaded surface (micro-grooved).

**Table 2:** Multiples comparisons: Type of surface and treatment, the number of adherent cells, and the mean (Standard error). Statistical analysis was performed by using three-way ANOVA with Tukey’s corrections for multiple comparisons. * represents a significant difference versus the relative time point control condition.

Surface	Treatment	Time	Fibroblasts
MAC	Control	20 min	37.13 (3.24)
		24 h	198.75 (8.85)
		72 h	372.75 (25.81)
	Plasma	20 min	75.38 (4.04) *
		24 h	211.25 (8.90)
		72 h	359 (21.82)
	UV	20 min	27 (3.30)
		24 h	207.62 (7.76)
		72 h	353.87 (30.09)
UTM	Control	20 min	29.25 (2.95)
		24 h	213.5 (9.18)
		72 h	379.12 (19.34)
	Plasma	20 min	65.88 (4.24) *
		24 h	203.37 (10.43)
		72 h	359.87 (20.66)
	UV	20 min	27.25 (3.44)
		24 h	206.37 (11.85)
		72 h	369.75 (19.67)
UTM-Y	Control	20 min	30.13 (3.60)
		24 h	205 (12.29)
		72 h	413.5 (29.95)
	Plasma	20 min	69.38 (5.02) *
		24 h	205.75 (18.13)
		72 h	431.37 (23.15)
	UV	20 min	28.75 (4.63)
		24 h	200.12 (10.45)
		72 h	364.25 (31.16)
XA	Control	20 min	32.50 (3.11)
		24 h	207 (10.97)
		72 h	479.25 (34.42)
	Plasma	20 min	68.75 (4.96) *
		24 h	204.25 (8.61)
		72 h	490 (35.45)
	UV	20 min	31.25 (3.09)
		24 h	208.25 (10.82)
		72 h	462.87 (30.94)

Surfaces (made of grade 5 Ti): MAC: machined titanium; UTM: ultrathin threaded microsurface titanium; UTM-Y: anodized ultrathin threaded microsurface titanium; XA: deep threaded surface. Treatments: control; no treatment; UV: ultraviolet light; plasma; non-thermal plasma treatment.

**Table 3:** Type of treatment, the number of adherent cells, and the mean (Standard error). Statistical analysis was performed by using three-way ANOVA with Tukey’s corrections for multiple comparisons. * represents a significant difference between the plasma treatment and the other groups of the relative time point.

Treatment	T0 20 min	T1 24 h	T2 72 h
Control	30.56 (1.16)	207.22 (3.91)	388.81 (12.78)
Plasma	72.06 (2.86) *	208.14 (4.43)	394.16 (12.34)
UV	29.93 (1.53)	209.92 (4.25)	379.41 (12.85)

**Table 4:** Type of surface, the number of adherent cells, and the mean (Standard error).Statistical analysis was performed by using three-way ANOVA with Tukey’s corrections for multiple comparisons.* represents a significant difference versus the relative time point MAC condition.

Surface	T0 20 min	T1 24 h	T2 72 h
MAC	37.13 (3.24)	198.75 (8.85)	372.75 (25.81)
UTM	29.25 (2.95)	213.5 (9.18)	379.12 (19.34)
UTM-Y	30.13 (3.60)	205 (12.29)	413.5 (29.95)
XA	32.50 (3.11)	207 (10.97)	479.25 (34.42)

Surfaces (made of grade 5 Ti): MAC: machined titanium; UTM: ultrathin threaded microsurface titanium; UTM-Y: anodized ultrathin threaded microsurface titanium; XA: deep threaded surface.

## Abbreviations

Ultraviolet light	UV
Machined	MAC
Ultrathin threaded microsurface	UTM
Anodized ultrathin threaded microsurface	UTM-Y
Thin machined	XA
Stereo scanning electron microscope	SSEM
Scanning electron microscope	SEM
Optical contact angle	OCA
Normal human dermal fibroblasts	NHDF
Dulbecco minimum essential medium	DMEM
Phosphate buffer saline	PBS
Paraformaldehyde	PFA
6-diamidino-2-phenylindole	DAPI
Focused ion beam	FIB
